# Sulfasalazine-Induced Epstein–Barr Virus-Positive Mucocutaneous Ulcer

**DOI:** 10.1155/2024/6883657

**Published:** 2024-07-02

**Authors:** Cedric Stabel, F. J. Sherida H. Woei-A-Jin, Thomas Tousseyn, Maria Garmyn

**Affiliations:** ^1^ Department of Dermatology University Hospitals Leuven, Leuven, Belgium; ^2^ Department of General Medical Oncology University Hospitals Leuven KU Leuven, Leuven, Belgium; ^3^ Department of Pathology University Hospitals Leuven, Leuven, Belgium

## Abstract

Epstein–Barr virus (EBV) may cause a wide spectrum of symptomatology in humans ranging from asymptomatic upper respiratory tract infection to infectious mononucleosis and in more severe cases lymphoproliferative disorders or hemophagocytic lymphohistiocytosis. Its neoplastic potential is higher in immunocompromised individuals. We describe a case of EBV-positive mucocutaneous ulcer, a more indolent clinical entity on the spectrum of EBV-driven lymphoproliferative disorders, and are one of the first to put sulfasalazine, an immunomodulatory agent, forward as the possible culprit.

## 1. Case Report

**Figure 1:**
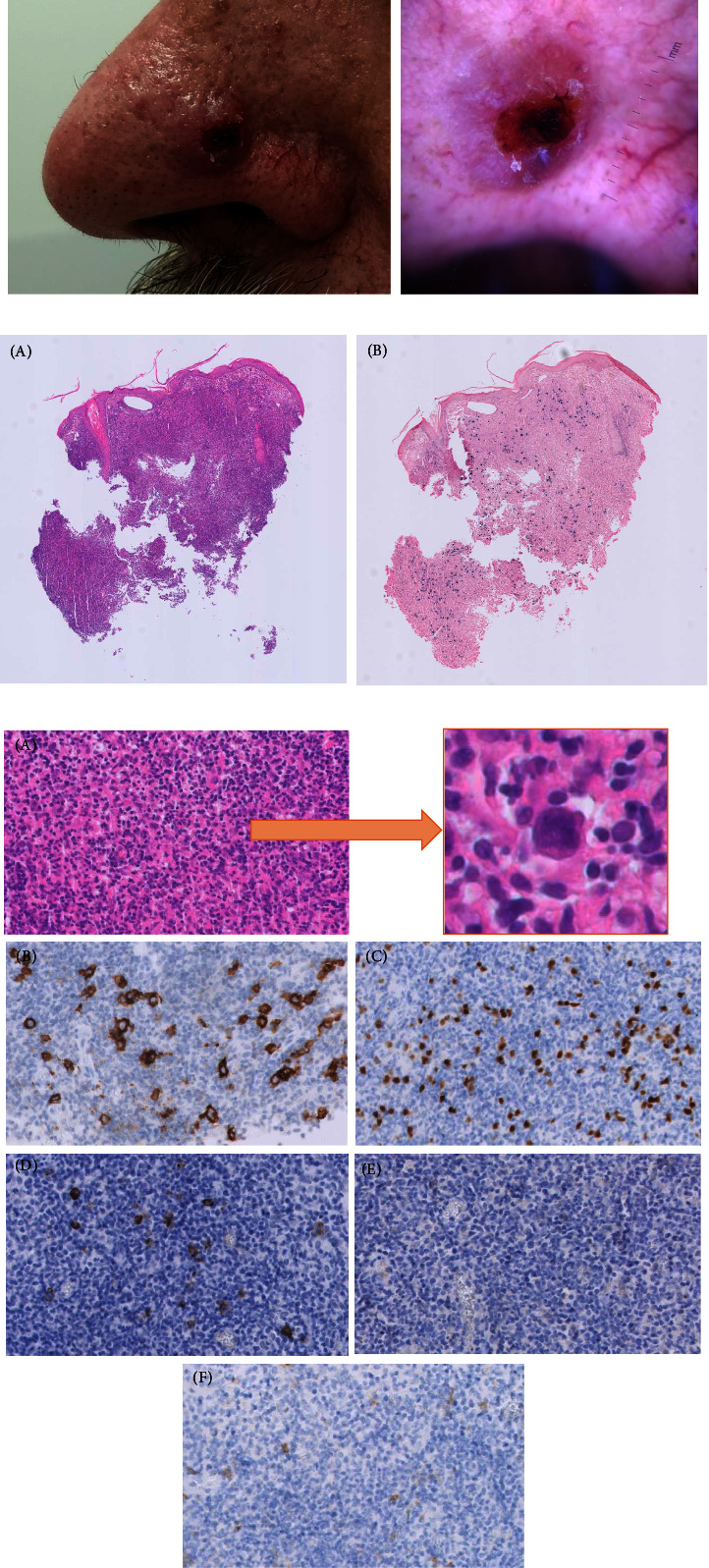
(a) (A) Clinical and (B) dermatoscopic picture (b) Low power views (25X): (A) HE shows a well-demarcated ulcerative skin lesion composed of a mixture of inflammatory cells and sparse EBV-transformed B-cells ((B). EBER ISH); and bordered at the periphery by small T-cells. (c) High power views (400X): (A) shows a polymorphic lymphoplasmacytic infiltrate with interspersed large atypical, Hodgkin/Reed–Sternberg-like cells (insert), expressing CD30 (B), PAX5 (C), EBV-LMP1 (D), but no EBNA2 (E), corresponding to an EBV latency type II. (F) The absence of NCAM/CD56 expression in the large EBV + cells excluded the possibility of an extranodal NK/T-cell lymphoma.

A 68-year-old male presented with a progressive nodular tumor on his left nostril, present for three months (Figure 1(a) (A)). His dermatologist suspected kerato-acanthoma, and the patient was referred for excision. The lesion arose spontaneously and grew without giving any complaints like pain, itch, or bleeding. B-symptoms were absent. It only started bothering him because of esthetical reasons.

His personal and familial history for cutaneous malignancy was negative, but he was known to have Crohn's disease, type II diabetes, hypertension, gout, and heartburn. Relevant chronic medication consists of respectively sulfasalazine, metformin, amlodipine, valsartan, bisoprolol, allopurinol, and omeprazole.

Clinically, the lesion was a papule, 1 cm in diameter, with a central ulceration covered by a necrotic crust. Upon palpation, the lesion was surprisingly soft and displayed dermatoscopically some inconspicuous telangiectasias with white streaks in its raised border (Figure 1(a) (B)). Besides its growth, these clinical findings and especially the softness were rather atypical for a kerato-acanthoma.

Initial punch biopsy showed a nonspecific chronic inflammation with a dense lympho-histiocytic inflammatory infiltrate without any atypia. As this did not correspond with clinical observations, a new larger fresh biopsy was performed, now also showing individual large Reed–Sternberg-like cells scattered in the infiltrate. EBV encoded RNA CISH (chromogenic in situ hybridization) was positive in a variety of B-lymphocytes, and polymerase chain reaction (PCR) showed the presence of a monoclonal immunoglobulin *κ* light chain gene and immunoglobulin heavy chain (Figures 1(b) and 1(c)). Clinicopathological correlation resulted in the diagnosis of an EBV-positive mucocutaneous ulcer (EBVMCU). Staging by ^18^F-FDG PET-CT revealed no other suspect lesions.

After stopping of sulfasalazine, which the patient has taken for multiple years at a dosage of 1 g twice daily, the lesion was clearly in regression at check-up after 3 weeks and fully disappeared after 50 days, which was confirmed by ^18^F-FDG PET-CT. There were no other medication changes in that time period. His Crohn's disease remained in persistent clinical remission up to this date, 3 years after discontinuation.

## 2. Discussion

Epstein–Barr virus (EBV), a member of the herpes virus family, is a ubiquitous cause of infection in humans. It is also known to cause a spectrum of lymphoproliferative disorders (LPDs) ranging from self-limiting local lesions to aggressive life-threatening systemic lymphomas such as classical Hodgkin lymphoma (cHL), diffuse large B-cell lymphoma (DLBCL), and natural killer (NK)/T-cell lymphoma, as well as solid malignancies such as nasopharyngeal carcinoma and smooth muscle tumors [1].

EBVMCU is a rare condition within the spectrum of EBV-driven LPDs with a slight male predominance. It mainly occurs in immunosuppressed (either primary, iatrogenic, or age-associated) patients and is thought to arise on sites subjected to tissue damage or inflammation [1]. It is defined as a solitary, sharply demarcated ulcerating lesion which localizes to the skin, oropharyngeal mucosa, or gastrointestinal tract [2]. Although regional lymphadenopathy may be present, systemic progression or relapse only occurs incidentally. Differential diagnosis in the nasal region includes extranodal natural killer/T-cell lymphoma, EBV-positive DLBCL, plasmablastic lymphoma, and anaplastic large cell lymphoma. Nonmelanoma skin cancers must always be in the differential diagnosis for cutaneous lesions. Histopathological differentiation between EBVMCU and DLBCL is challenging. Thorough clinicopathologic investigation and clinical follow-up are thus warranted not to miss this more aggressive lymphoma.

Regression of an EBVMCU can occur spontaneously in age-associated immunosenescence or drug-related immunosuppression, though mostly requires a dose reduction or switch of immunosuppressive therapy.

In our patient, we hypothesized that the immunomodulatory agent sulfasalazine (=salazosulfapyridine) was the culprit considering the *in vitro* inhibition of transcription factor Nuclear Factor kappa B, which plays an essential role in the regulation of immune responses and inflammation, the inhibition of T-cell proliferation, and alteration of cytokine profiles affecting T-cell cytokines IL-2 and interferon-*γ* [3–5]. Only after the patient stopped, slow regression of the nodule was noted.

This is the first report describing remission of an EBVMCU after sulfasalazine withdrawal, although recently remission of DLBCL following sulfasalazine withdrawal in a patient with rheumatoid arthritis was described [6]. Prieto-Torres et al. described development of a gastrointestinal EBVMCU in an inflammatory bowel disease patient who was being treated with mesalazine. Sulfasalazine and mesalazine both are anti-inflammatory agents from the 5-aminosalicylic acid group. Mesalazine was not stopped in this patient, and the lesion persisted on colonoscopy after 6 months, although associated symptoms of rectal bleeding subsided [7]. Based on our observations, mesalazine could have been a culprit of the EBVMCU and it would have been interesting to see whether a stop would result in complete regression.

Sulfasalazine can also cause a drug-induced hypersensitivity syndrome (defined as reactions resulting from unintended stimulation of immune cells) and in this context give rise to EBV reactivation as described by Komatsuda et al. [8]. Their patient subsequently also developed a hemophagocytic syndrome. Drug-induced hypersensitivity however generally occurs in a much shorter time period (12 weeks) after introduction of sulfasalazine; thus, the time frame of this disease entity does not fit the disease course in our patient.

Spontaneous regression of EBVMCU in an individual with age-related immunosuppression is reported to occur in ∼55% of elderly patients not receiving immunosuppressive agents [9]. There are, however, reports of a more persistent and debilitating course that require surgical excision, radiation therapy, or systemic immunochemotherapy therapy such as anti-CD20 monoclonal antibody rituximab. Multiagent chemotherapy is seldom indicated [9]. In one refractory case, anti-CD30 antibody-drug conjugate brentuximab-vedotin was administered resulting in complete remission [10].

## Data Availability

The data used to support the findings of this study are available from the corresponding author upon reasonable request.
